# Direct electrochemical measurement of metanephrines in spot urine samples for the diagnosis of phaeochromocytomas

**DOI:** 10.1038/s41598-017-08612-8

**Published:** 2017-08-14

**Authors:** Zheng-Hu Shi, Xiao-Qing Zhang, Qian-Na Zhen, Ming Zuo, Gang Tian, Yi-Fan He, Min Ding

**Affiliations:** 1 Key Laboratory of Clinical Laboratory Diagnostics (Ministry of Education of China), School of Laboratory Medicine, Chongqing Medical University, Chongqing, 400016 P. R. China; 2 Department of Endocrinology, the First Affiliated Hospital of Chongqing Medical University, Chongqing, 400016 China

## Abstract

Metanephrines (MNs) were suggested as a potential first-line biochemical index for the diagnosis of phaeochromocytomas (PHEO). In this study, we developed a simple electrochemical method for the quantitative measurement of MNs in spot urine samples. As MNs contain a hydroxyphenyl group, they could be oxidized at a certain potential to quinines, which could be further detected by the differential pulse voltammetry (DPV) method using unmodified screen-printed carbon electrode (SPCE). Meanwhile, the solid phase extraction (SPE) technique was used to eliminate the matrix effect in the samples. Consequently, free MNs from the extracted urine sample were screened in a linear range from 0.25 mg/L to 12.5 mg/L. The lowest limit of quantification (LLOQ) for MNs was 0.25 mg/L and the limit of detection (LOD) was 0.05 mg/L. Both the precisions and recoveries were sufficient for clinical applications. The urine samples from 22 patients with PHEO and 63 controls were analyzed by the proposed method. The area under the ROC curve was 0.981 (95% CI, 0.958–1.000) with the sensitivity of 95.5% and the specificity of 92.4% at the cut-off value of 0.404 mg/L in these urine samples. Overall, the proposed method provides a cost-effective, rapid and simple tool for clinical diagnosis of PHEO.

## Introduction

Phaeochromocytomas (PHEO) are neuroendocrine tumors arising from chromaffin cells and characterized by excessive production of catecholamines (CAs) and their metabolites^1–3^. These tumors have fatal consequences due to the potential impact of these compounds on the cardiovascular system^4, 5^. Therefore, it is crucial for the patients with PHEO to be correctly diagnosed with such tumors so that they can be timely treated. Metanephrines (MNs, including metanephrine and normetanephrine), the O-methylated extraneuronal metabolites of CAs, are the first-line biochemical indices currently used for the diagnosis of suspected PHEO^1, 2, 6^.

Several methods such as enzyme-linked immunosorbent assay (ELISA)^7, 8^, radioimmunoassay (RIA)^9^, high performance liquid chromatography with mass spectrometry (HPLC-MS)^10–12^ and electrochemical detection (HPLC-ED)^13, 14^ have been well established for the quantification of MNs in human plasma or urine. Among these methods, ELISA is the most commonly used method in clinics. Very often the cross-talk and non-specific binding in ELISA may lead to erroneous results. The RIA technique is not currently applied in the typical hospital setting due to its potential radioactive contaminations. Although both HPLC-MS and HPLC-ED are sensitive in the measurement, expensive instruments and experienced operators are required for these methods. Owing to the limitations of the above-mentioned analytical methods, researchers continuously strive to explore a simple alternative analytical technique that can be readily applied to the measurement of clinical samples. Electrochemical detection technique is an extremely promising alternative method that allows for real-time, in/ex-*situ* monitoring of the metabolites in a variety of biological samples^15, 16^. Furthermore, the use of screen-printed carbon electrodes (SPCEs) renders the electrochemical detection technique several additional advantages such as easy and cost-effective electrode fabrication and capablility of mass production, which makes it attractive for clinical diagnostic applications^17–20^.

In this study, a simple electrochemical detection method was developed using unmodified SPCEs for the quantitation of MNs in human spot urine samples. As MNs contain a hydroxyphenyl group, they can be readily oxidized to quinines at a certain potential. The electron transfer in such process can be conveniently measured^21^. Meanwhile, the solid phase extraction (SPE) technique was used to eliminate the matrix effect of these urine samples to reduce the background interferences. Consequently, the concentration of MNs in human spot urine samples can be detected by differential pulse voltammetry (DPV). The proposed method was successfully applied to determine urinary MNs in PHEO patients in comparison with the control groups. Receiver operating characteristic (ROC) curves were further used to assess the performance of urinary MNs for the diagnosis of PHEO. This method is accurate and cost-effective. Moreover, it has potential to be used for clinical diagnosis of PHEO.

## Results and Discussion

### Principle of electrochemical detection of MNs

**Figure 1:**
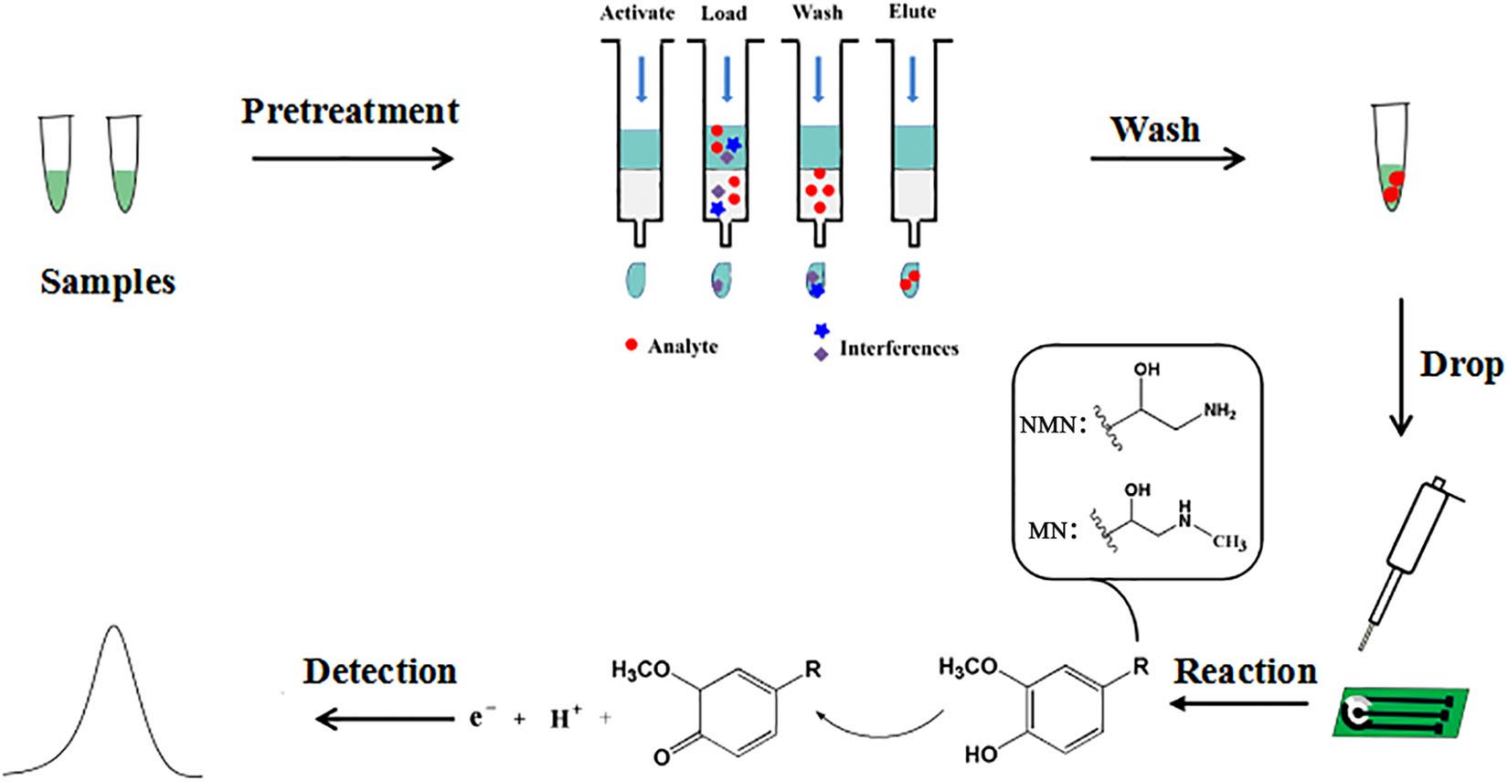
Schematic illustration of the experimental procedure performed on the SPCE.

MNs (MN and NMN) are the O-methylated extraneuronal metabolites of CAs, which contain a hydroxyphenyl group as a common structure element. Although NMN and MN have different groups for R in Fig. 1, the hydroxyphenyl group can be oxidized to quinine at a certain potential, which causes the flow of electrons creates measurable current signals. Consequently, these signals were detected by DPV method. Based on our experimental data (Supplementary Figure S1), MN and NMN exhibited a similar potential window in buffer solution. Thus, we detected a mixture of MN and NMN. The result showed that the oxidation peaks of MN and NMN appeared at the potential of 0.35 V. The experimental procedure including the principle of the method was illustrated in Fig. 1.

### Matrix effects

**Figure 2:**
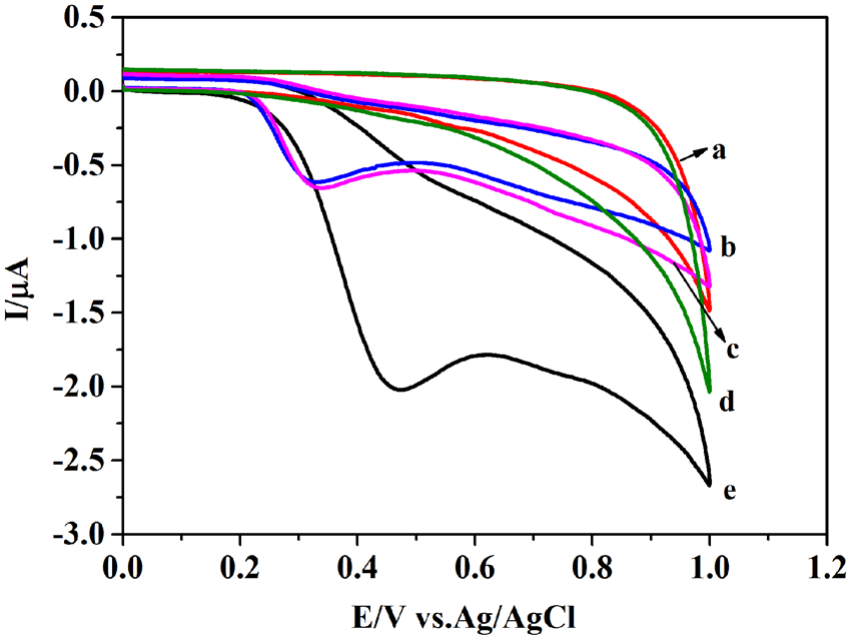
CV curves of blank urine after solid phase extraction (SPE) (a) Spiked MNs (10.0 mg/L) in mixed blank urine after SPE (b) Spiked MNs (10.0 mg/L) in PBS (c) 100 mM PBS (pH8.0) (d) Blank urine (e).

To investigate the matrix effects in urine, strong cation exchange resin SPE was used as sample preparation in this work. Phosphate buffered saline (PBS, 100.0 mM, pH 8.0), PBS spiked with MNs (10.0 mg/L), blank urine, blank urine after SPE and blank urine spiked with MNs (10.0 mg/L) after SPE were measured by cyclic voltammetry (CV), respectively (Fig. 2). The peak potential and peak current in the urine spiked with MNs after SPE were similar to that in PBS spiked with MNs, as shown in curve b, c (Fig. 2). However, the blank urine generated a large background current at the peak potential of 0.50 V, given in curve e (Fig. 2). These results indicated that matrix effects of urine interfered with the oxidation of MNs at the surface of the electrodes, which caused higher peak potential and background current. Consequently, strong acidic cation exchange resin SPE was adopted in the subsequent work to eliminate the matrix effects in urine.

### Optimization of the experimental conditions

**Figure 3:**
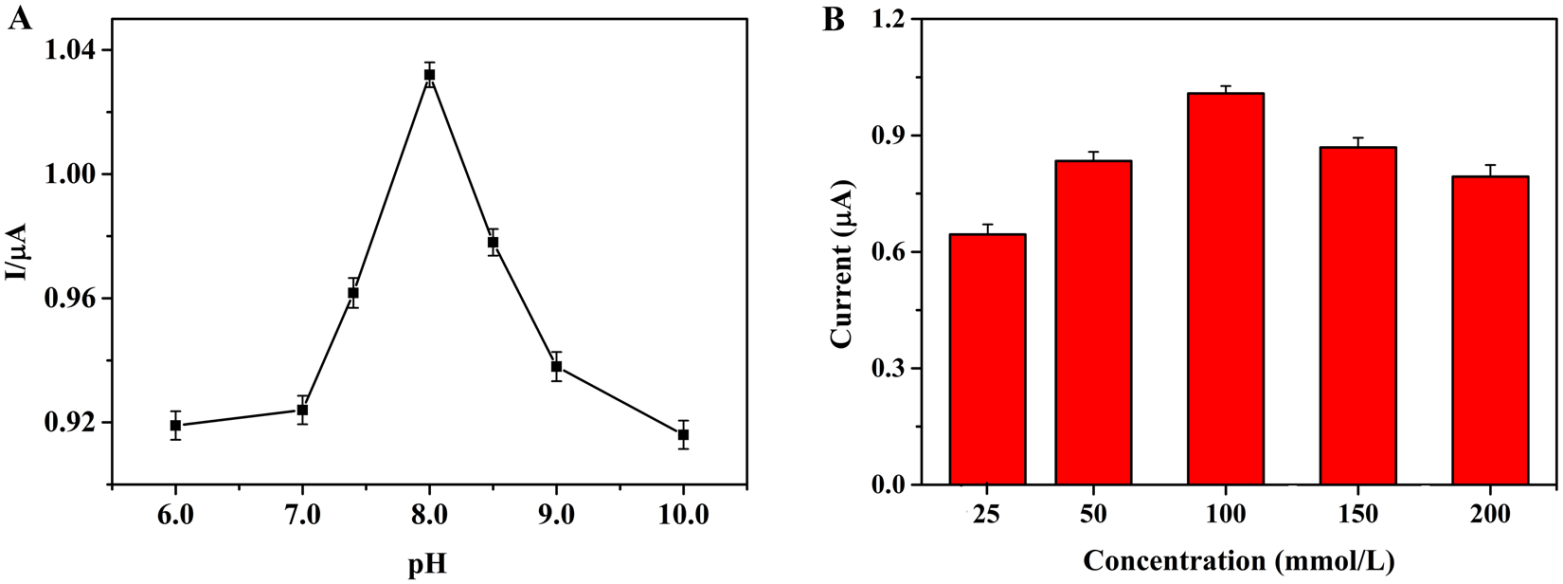
Optimizations of experimental parameters: (**A**) pH value, (**B**) Concentration of PBS. Error bars are standard deviations.

The supporting electrolyte conditions (pH and concentration) are key factors which impact on the electrochemical activity of MNs. A plot of current responses in 100 mM PBS containing MNs standard solution (10.0 mg/L) with different pH values, shown in Fig. 3A. The results showed that the current response increased obviously with the increase of pH value from 6.0 to 8.0, and then reached to a maximum value at pH 8.0. The current response decreased as the pH value was over 8.0. In addition, the baseline of the current response was much stable and the noise was low at pH 8.0 in blank urine. With the increase of the concentration of PBS, the response signal increased and reached to the highest value at 100 mM and then it gradually declined until at 200 mM, given in Fig. 3B. Therefore, 100 mM PBS pH 8.0 was selected as the working buffer in the following experiments.

## Method validation

### Calibration curve and linearity

**Figure 4:**
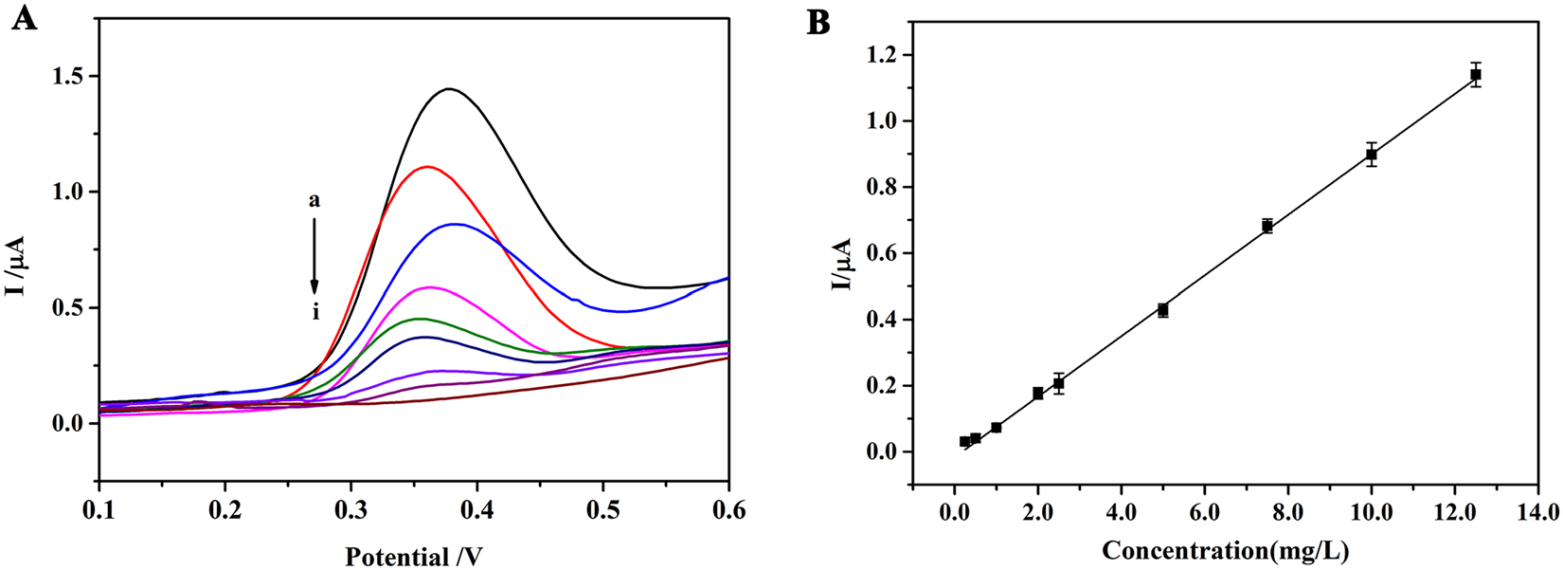
The linearity of the method. (**A**) Typical DPV recorded over a range of MNs concentrations (a–i, 0.25 to 12.5 mg/L) in spiked mixed blank urine sample using the standard addition method. (**B**) The corresponding calibration plots for different concentrations of MNs (0.25, 0.5, 1.0, 2.0, 2.5, 5.0, 7.5, 10.0, 12.5 mg/L). Error bars are standard deviations and supporting electrolyte: 100 mM PBS (pH 8.0).

Under the optimal experimental conditions, different concentrations of MNs were added into the blank urine to prepare the standard working solutions for the electrochemical determination. The DPV curves for different concentrations of MNs were displayed in Fig. 4A. The calibration curve of MNs showed the linearity at the concentration ranging from 0.25 to 12.5 mg/L in urine, shown in Fig. 4B. The average regression equation for the calibration curves could be expressed as *Y* = 0.916 *X*- 0.127 (r = 0.994, n = 3). According to the guideline of the Clinical and Laboratory Standards Institute (CLSI) in clinical chemistry (EP17-A), low concentration of analyte in the samples was determined and then the stepwise dilution was performed and ultimately the minimum level at which the analyte can be quantified was established as LLOQ. The LLOQ for MNs was defined as the lowest concentration in the linear range of the calibration curves and the relative standard deviation (RSD) was less than 20%. The LLOQ for MNs was 0.25 mg/L. Based on signal-to-noise approach, the LOD for MNs was 0.05 mg/L (S/N = 3).

### Precision and recovery

Precisions were evaluated by assaying five replicates at low, medium and high concentrations of the standard working solutions (0.5, 5.0, 10.0 mg/L) on the same day (intra-day) and on five consecutive days (inter-day). Precision was expressed by the RSD. The RSD for intra-day precisions were ranged from 5.9% to 9.7% and RSD for inter-day precisions were ranged from 6.8% to 9.9%. The intra-day and inter-day precisions of MNs at LLOQ were 9.9% and 10.2%, respectively. The precisions were acceptable for the determination of clinical samples, as listed in Table 1. Recovery tests were determined by measuring the concentrations of MNs in 990 μL of blank urine before and after adding 10 μL of standard solutions of MNs at low (0.5 mg/L), medium (5.0 mg/L) and high concentrations (10.0 mg/L). The recoveries for different concentrations of MNs were ranged from 82.0% to 101.1% for intra-day, and 81.0% to 96.5% for inter-day, respectively. The intra-day and inter-day recoveries of MNs at LLOQ were 81.0% and 80.5%, respectively. It indicated an acceptable recovery.

**Table 1:** The precisions and recoveries of MNs detected by the proposed method in spot urine samples (n = 5).

Added concentration (mg/L)	Measured concentration (Mean ± SD, mg/L)	Precision (RSD, %)	Recovery (%)
Intra-day			
0.50	0.410 ± 0.040	9.7	82.0
5.0	4.71 ± 0.42	8.9	94.2
10.0	10.11 ± 0.60	5.9	101.1
Inter-day			
0.50	0.405 ± 0.040	9.9	81.0
5.0	4.64 ± 0.43	9.2	92.8
10.0	9.65 ± 0.66	6.8	96.5

### Interference test

Ascorbic acid (AA), uric acid (UA), creatinine (Cr) and urea are usually defined as endogenous interferences in human urine. Interference test was performed according to the guideline of the Clinical and Laboratory Standards Institute (CLSI-EP7-A) for clinical chemistry. The concentrations of MNs in urine before and after adding standards of interferences were defined as *X*_C_ and *X*_T_, respectively. The interference value, expressed as (*X*_T_ − *X*_C_), less than 1.96 *S* showed insignificant interference, which was expressed by N. While the interference value more than 1.96 *S* indicated significant interference, which was expressed by I. The results showed that there was insignificant interference when the concentration of AA, Cr, urea and UA at the recommended test concentrations, shown in Table 2. Therefore, the proposed method possessed anti-interference capability for the determination of MNs in urine samples.

**Table 2:** The effect of interferences on the determination of MNs (n = 5).

Added interferences (mg/L)		Concentration of MNs (mg/L)		
		Measured	*X*_T_ − *X*_C_	1.96 *S*
Blank	0.0	2.43	—	0.32
Urea	250.0	2.38	−0.05	N
	500.0	2.20	−0.23	N
Creatinine	50.0	2.44	0.01	N
	100.0	2.29	−0.14	N
Ascorbic acid	50.0	2.33	−0.10	N
	100.0	2.39	−0.04	N
Uric acid	50.0	2.32	−0.11	N
	100.0	2.42	−0.01	N

### Clinical application

**Figure 5:**
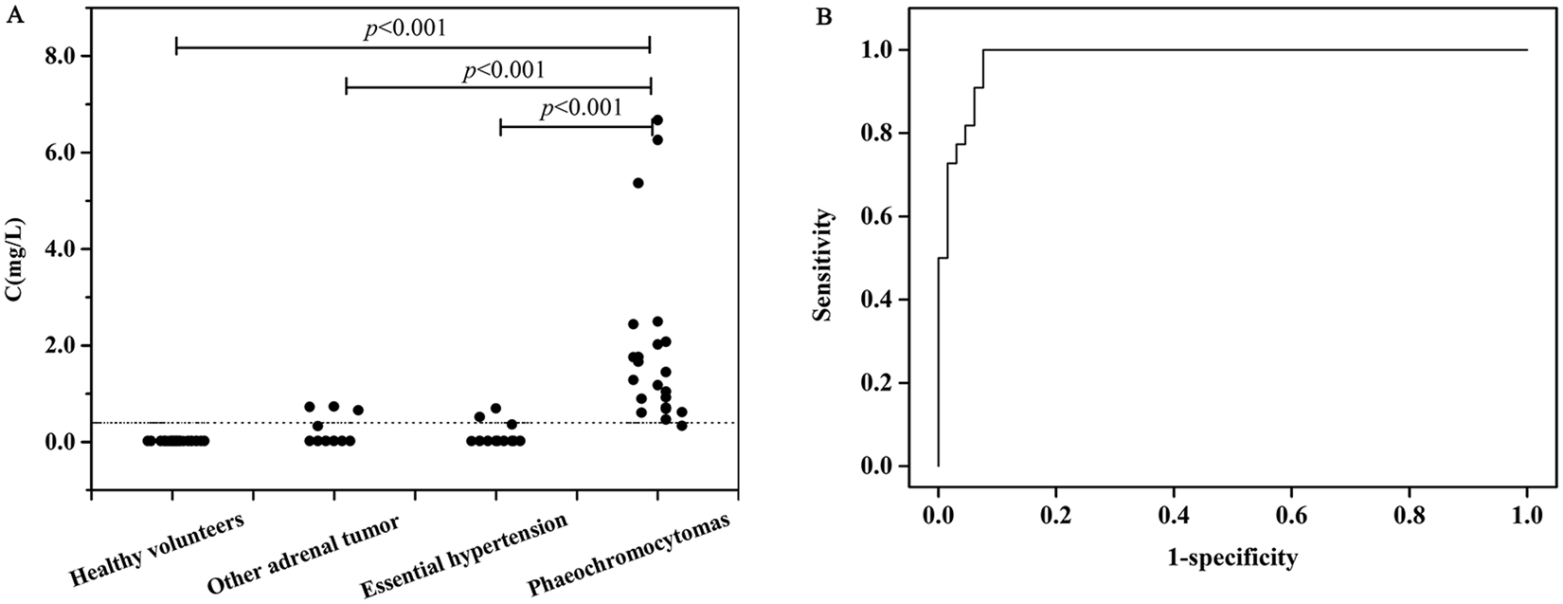
Diagnostic efficacy for detection of PHEO. (**A**) Distribution of free MNs concentrations in spot urine of subjects in this study. (**B**) ROC curves for the diagnostic performance of spot urinary MNs. Dash dotted line, cut-off value.

Spot urinary samples from PHEO patients and controls were analyzed by the proposed method. The results for free urinary MNs are shown in Fig. 5A. The concentration of MNs in patients with PHEO was significantly higher than that in the three control groups (*p* < 0.001). However, the concentration of MNs had no significant differences among the control groups. The median level of MNs in the patients with PHEO was 1. 37 mg/L (0.34~6.67 mg/L). ROC curve was employed to assess optimal diagnostic efficacy. As shown in Fig. 5B, the area under the ROC curve was 0.981 (95% CI, 0.958–1.000) with 95.5% of sensitivity and 92.4% of specificity at the cut-off value of 0.404 mg/L in spot urine. The concentration of MNs of PHEO patients samples obtained by the proposed method were significantly correlated with MNs measured by the reference method of HPLC-ED^22^ (r = 0.941, *p* = 0.000) in Supplementary Figure S2.

## Conclusions

A novel electrochemical method for the quantitative measurement of MNs in spot urine samples was established with unmodified SPCE. This method was successfully used for the measurement of MNs in clinical samples. Using clinically derived cut-offs for positivity demonstrated a high diagnostic efficacy of MNs in spot urine samples. This work developed a simple, cost-effective electrochemical method for the determination of MNs in spot urine samples for clinical diagnostic applications. For future prospective, our work will be focused on simplifying the process for sample pretreatment to reduce the analysis time and multicenter trials patients are required to confirm our findings.

## Methods

### Materials and reagents

Metanephrine (MN) was purchased from Shanghai Binglingwei Co., Ltd. (Shanghai, China). Normetanephrine (NMN) was obtained from Sigma-Aldrich. Na_2_HPO_4_ and NaH_2_PO_4_ were obtained from Kelong Chemical Company (Chengdu, China). All other reagents were of analytical grade and obtained from Shanghai Company of Chemical Reagent (Shanghai, China). Solid phase extraction (SPE) cartridges (SampliQ Silica SCX, 100 mg/1 mL) were purchased from Agilent (Agilent, USA). The ultra-pure water was obtained from a Milli-Q water purification system (Millipore, Bradford, USA). The electrochemical measurements were performed on CHI 852 C electrochemical workstation (Shanghai Chenhua Instruments Co. Ltd., China). The screen printed carbon electrode (3 mm in diameter) consists of three electrodes: A printed carbon working electrode, a printed carbon counter electrode and a printed Ag/AgCl reference electrode.

The stock solutions of MN and NMN were prepared by dissolving standard samples in 0.2 mol/L acetic acid and stored at −20 °C. Standard solution of MNs was prepared by mixing MN and NMN at the same concentration.

### Samples collection

22 patients with PHEO and 63 controls were enrolled from August 2014 to April 2016 in this study. The controls consisted three groups. The first group incorporated 20 patients with a clinical presentation suggestive of PHEO (essential hypertension difficult to treat with recent onset of headaches, sweating, or pallor paroxysms). The second group included 20 patients screened for PHEO after the discovery of other adrenal tumor mass (tumor-like adrenal image on abdominal computed tomography scan or ultrasound requested for unrelated reasons). The last group was composed of 23 healthy volunteers. The gold standard for the confirmation of PEHO was histopathology. All these patients were checked in a multidisciplinary clinic involving endocrinologists, nuclear medicine specialists and endocrine surgeons. This study was performed at the First Affiliated Hospital of Chongqing Medical University, Chongqing, and designed conformed to the ethics guidelines given in the Declaration of Helsinki. Written informed consent was obtained from all subjects and all experimental protocols were approved by the ethics committee of the First Affiliated Hospital of Chongqing Medical University.

Subjects were instructed to fast or abstain from caffeinated and decaffeinated beverages overnight and avoid taking acetaminophen 5 days before sampling. All specimens were collected and analyzed by the same investigator blinded to the clinical condition of the patients. Spot urine samples were collected in 10 mL plastic centrifuge tube and kept at −80 °C before use. Urine with insufficient volume were excluded. 20 clinical spot urine samples with low concentrations of MNs (<0.10 mg/L) were mixed and used as a blank urine to complete the establishment and evaluation of the experimental method.

### Samples preparation

The sample preparation method was based on Yang and coworkers’ work^22^. 200 μL of urine was diluted with 5 mL of ultrapure water, and then 200 μL of 0.2 mol/L acetic acid was added. The diluted sample was loaded on the SPE cartridges previously conditioned with 5 mL of 10% ammonium and methanol (1:3, v/v, ammoniacal methanol), and 2 mL of 10 g/L KOH in methanol, followed by 2 mL ultrapure water. The cartridges were washed with 2 mL of 10 mmol/L acetic acid-methanol (9:1, v/v), 2 mL of 10 mmol/L ammonium phosphate and 2 mL ultrapure water, respectively. The analytes were eluted with 2 mL of ammoniacal methanol. The eluate was dried by vacuum centrifugation and reconstituted with 200 μL of 0.2 mol/L acetic acid.

### Validation of the electrochemical method

Different concentrations of MNs were added into blank urine to prepare the standard working solutions for the electrochemical determination. The linearity of the calibration curves (ranging from 0.25 to 12.5 mg/L) was assessed based on nine sets independently prepared working standard solutions. LOD was defined as the lowest concentration with a signal-to-noise ratio greater than three, and LLOQ was determined as the lowest concentration of linearity to be measured within 20% of RSD. Precisions were evaluated by assaying five replicates at three concentrations of the standard working solutions (0.5, 5.0, 10.0 mg/L) on the same day (intra-day) and on five consecutive days (inter-day). Recovery tests were determined by measuring the concentrations of MNs in 990 μL blank urine before and after adding 10 μL of standard solutions of MNs at low (0.5 mg/L), medium (5.0 mg/L) and high concentrations (10.0 mg/L).

According to the EP7-A guideline, interfering substances at high test concentrations are given for AA at 40.0 mg/L, Cr at 50.0 mg/L, urea at 400.0 mg/L and UA at 90 mg/L, respectively. To evaluate the selectivity of this assay, different concentrations of AA (50.0 and 100.0 mg/L), Cr (50.0 and 100.0 mg/L), urea (250.0 and 500.0 mg/L) and UA (50.0 and 100.0 mg/L) were added into the blank urine with spiked MNs (2.5 mg/L).

### Electrochemical measurement

25 µL of urine sample after pretreatment was added into 25 µL of PBS (100.0 mM, pH 8.0) and mixed completely. Then, 50 µL of the mixed solution was added to the working electrode on the SPCE, which was connected to the electrochemical workstation. Subsequently, the electrochemical signal was detected by DPV. The detection voltage was set from 0 V to 1.0 V with the pulse amplitude of 50 mV. The pulse width was set at 0.05 s. And the pulse period was set at 0.2 s.

### Statistical Analysis

For the “Non-detects” data in the control group, the results were set as one half of the LOD for statistical analysis. All the statistical analysis were performed by SPSS Statistics. V20.0 (SPSS, Inc.,Chicago, USA). *p* value less than 0.05 was considered statistically significant.

## Electronic supplementary material

supporting information
