# Supplementary material for: Direct electrochemical measurement of metanephrines in spot urine samples for the diagnosis of phaeochromocytomas
Source: Sci Rep. 2017 Aug 14;7:8041. doi: 10.1038/s41598-017-08612-8 (PMC5556028; doi:10.1038/s41598-017-08612-8)
Supplement: Supplementary file 1 — supporting information [file 41598_2017_8612_MOESM1_ESM.pdf]

Supporting Information

Direct electrochemical measurement of metanephrines in spot urine samples for the diagnosis of phaeochromocytomas

Zheng-Hu Shi[1*], Xiao-Qing Zhang[1*], Qian-Na Zhen[2], Ming Zuo[1], Gang Tian[1], Yi-Fan He[1]& Min Ding[1]

[1]Key Laboratory of Clinical Laboratory Diagnostics (Ministry of Education of China), College of Laboratory Medicine, Chongqing Medical University, Chongqing, 400016, P. R. China. Correspondence and requests for materials should be addressed to M.D. (email:dingmin@ cqmu.edu.cn)

[2]Department of Endocrinology, the First Affiliated Hospital of Chongqing Medical University, Chongqing 400016, China

[*]These authors contributed equally to this work.

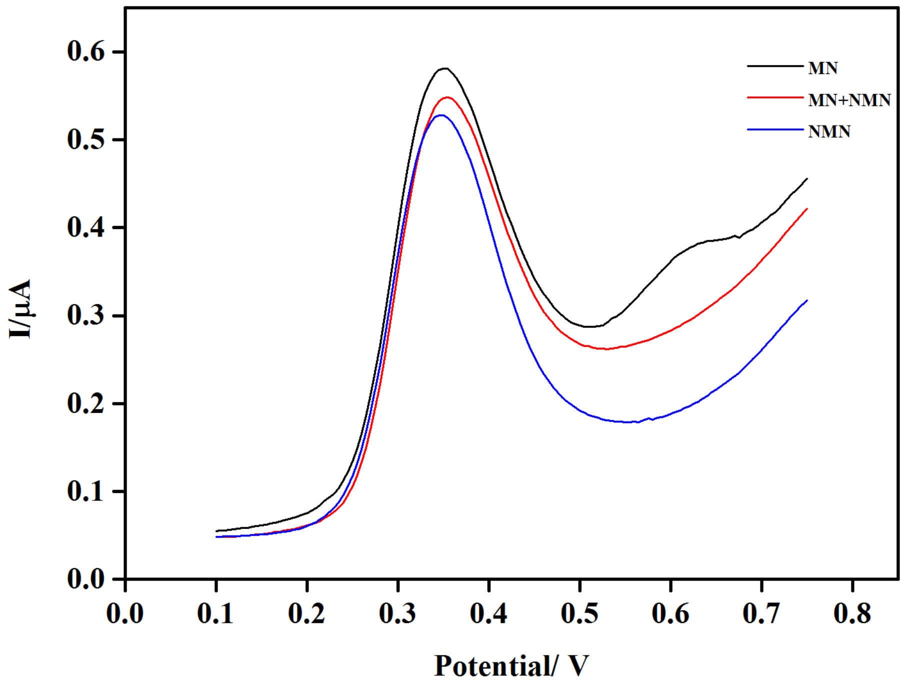

Figure S1. The DPV curves for NMN (10.0 mg/L) (Blue), MN (10.0 mg/L) (Black) and MN+NMN (NMN: 5.0 mg/L, MN: 5.0 mg/L) (Red).

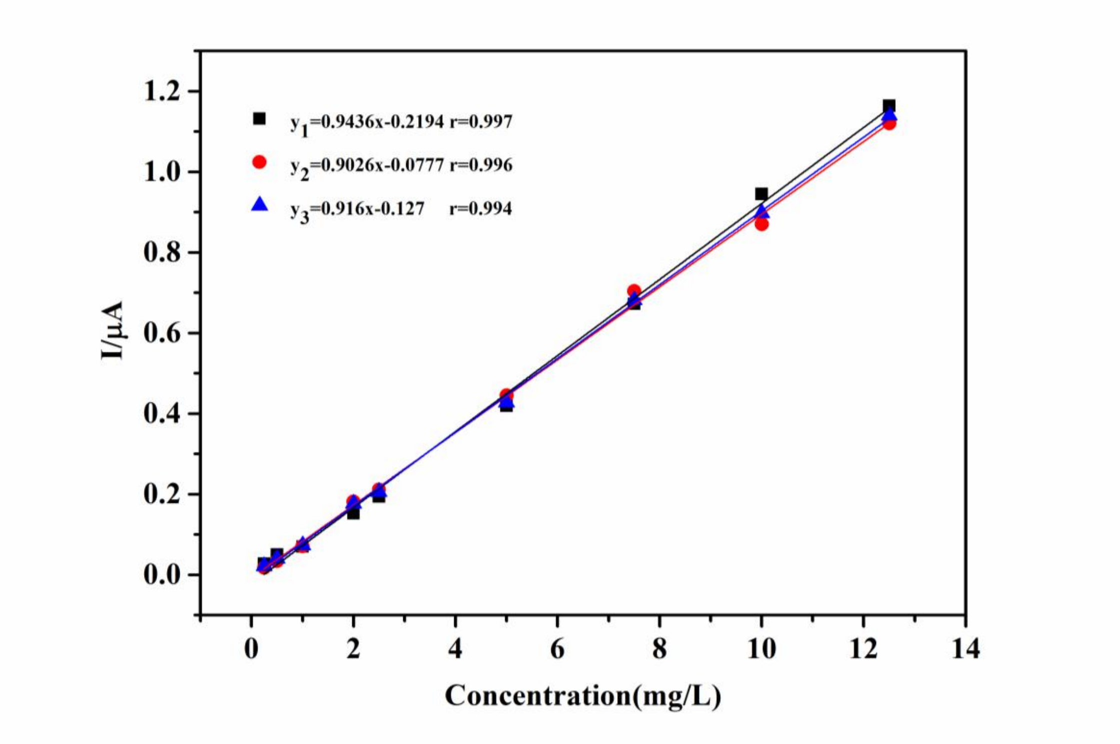

Figure S2. The calibration curves for MN (Dot)，NMN (Square) and MN+NMN (Triangle), respectively, (0.25, 0.5, 1.0, 2.0, 2.5, 5.0, 7.5, 10.0, 12.5) mg/L.

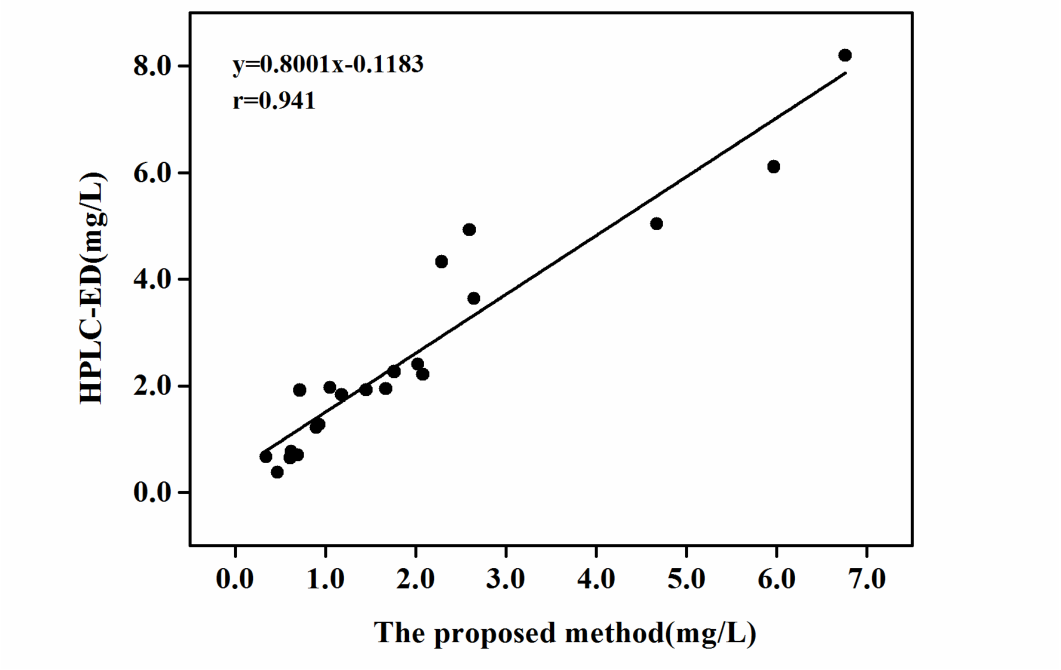

Figure S3. Correlation of MNs concentrations in the urine of PHEO patients measured by the established method and the reference method of HPLC-ED (r=0.941, p=0.000).
